# Characterization of Shiga toxin-producing *Escherichia coli* isolated from healthy pigs in China

**DOI:** 10.1186/1471-2180-14-5

**Published:** 2014-01-06

**Authors:** Qiong Meng, Xiangning Bai, Ailan Zhao, Ruiting Lan, Huamao Du, Tao Wang, Changyou Shi, Xuejiao Yuan, Xuemei Bai, Shaobo Ji, Dong Jin, Bo Yu, Yan Wang, Hui Sun, Kai Liu, Jianguo Xu, Yanwen Xiong

**Affiliations:** 1 State Key Laboratory for Infectious Disease Prevention and Control, National Institute for Communicable Disease Control and Prevention, Chinese Center for Disease Control and Prevention, Changping, Beijing, China; 2 Collaborative Innovation Center for Diagnosis and Treatment of Infectious Diseases, Hangzhou, China; 3 School of Biotechnology and Biomolecular Sciences, University of New South Wales, Sydney, NSW 2052, Australia; 4 Biochemical and Molecular Biological Department, School of Biotechnology, Southwest University, Chongqing, China; 5 Department of Microbiology, School of Basic Medical Sciences, Guiyang Medical University, Guiyang, China; 6 Animal Nutrition Institute, Sichuan Agricultural University, Chengdu, China

**Keywords:** Shiga toxin-producing *Escherichia coli* (STEC), Shiga toxin, Multilocus sequence typing, Adhesin genes, Putative virulence genes, Antibiotic resistance, Pulsed-field gel electrophoresis, Swine

## Abstract

**Background:**

Shiga toxin-producing *Escherichia coli* (STEC) is recognized as an important human diarrheal pathogen. Swine plays an important role as a carrier of this pathogen. In this study we determined the prevalence and characteristics of STEC from healthy swine collected between May 2011 and August 2012 from 3 cities/provinces in China.

**Results:**

A total of 1003 samples, including 326 fecal, 351 small intestinal contents and 326 colon contents samples, was analyzed. Two hundred and fifty five samples were *stx*-positive by PCR and 93 STEC isolates were recovered from 62 *stx*-positive samples. Twelve O serogroups and 19 O:H serotypes including 6 serotypes (O100:H20/[H20], O143:H38/[H38], O87:H10, O172:H30/[H30], O159:H16, O9:H30/[H30]) rarely found in swine and ruminants were identified. All 93 STEC isolates harbored *stx*_2_ only, all of which were *stx*_2e_ subtype including 1 isolate being a new variant of *stx*_2e_. 53.76%, 15.05% and 2.15% STEC isolates carried *astA*, *hlyA* and *ehxA* respectively. Four STEC isolates harbored the high-pathogenicity island. Of the 15 adherence-associated genes tested, 13 (*eae*, *efa1*, *iha*, *lpfA*_O113_, *lpfA*_O157/OI-154_, *lpfA*_O157/OI-141_, *toxB*, *saa*, F4, F5, F6, F17 or F41) were all absent while 2 (*paa* and F18) were present in 7 and 4 STEC isolates respectively. The majority of the isolates were resistant to tetracycline (79.57%), nalidixic acid (78.49%), trimethoprim-sulfamethoxazole (73.12%) and kanamycin (55.91%). The STEC isolates were divided into 63 pulsed-field gel electrophoresis patterns and 21 sequence types (STs). Isolates of the same STs generally showed the same or similar drug resistance patterns. A higher proportion of STEC isolates from Chongqing showed multidrug resistance with one ST (ST3628) resistant to 14 antimicrobials.

**Conclusions:**

Our results indicate that swine is a significant reservoir of STEC strains in China. Based on comparison by serotypes and sequence types with human strains and presence of virulence genes, the swine STEC may have a low potential to cause human disease.

## Background

*Escherichia coli* that produces one or more types of cytotoxins known as Shiga toxin (Stx) or Verocytotoxin (VT) is referred to as Shiga toxin-producing *E. coli* (STEC) or Verocytoxion-producing *E. coli* (VTEC) [1]. STEC is a well-known pathogen as a cause of diarrhea, hemorrhagic colitis (HC) and hemolytic uremic syndrome (HUS) [2]. Most cases of HC and HUS have been attributed to STEC O157:H7, but the importance of non-O157 STEC is increasingly recognized [3].

STEC possesses a number of virulence factors. Besides the *stx* genes, human pathogenic STEC strains often carry the *eae* gene, one of the genes located on LEE pathogenicity island encoding the adherence factor intimin [4] and the *astA* gene encoding a heat-stable enterotoxin EAST1 [5]. STEC strains may also be hemolytic due to the presence of the α-hemolysin or the enterohemolysin or both. The α-hemolysin gene *hlyA* is located on the chromosome [6] while the enterohemolysin (*ehxA*) is harbored by a plasmid [7]. Many adherence-related factors were found in STEC [8-13]. EHEC factor for adherence (*efa1*) was shown to be essential for the adherence of the bacteria to cultured epithelial cells [11]. The IrgA homologue adhesin (*iha*) is a STEC adherence-conferring molecule conferring the adherence phenotype upon a nonadherent laboratory *E. coli* strain [13]. *lpfA*_O113_, *lpfA*_O157/OI-154_ and *lpfA*_O157/OI-141_ are adhesion genes in LEE-negative STEC strains [9,14]. Many STEC strains contain the heterologous 60-MDa virulence plasmid, which encodes a potential adhesin ToxB [10]. Other novel adhesion factors reported include autoagglutinating adhesin (*saa*) [12] and porcine attaching and effacing (A/E) associated protein (*paa*) [8]. Most STEC strains isolated from diarrheal pigs can produce one or more of the fimbriae, F4, F5, F6, F17, F18 and F41. Different types of fimbriae were reported to be associated with STEC diarrhea in animals of different age groups [15-18]. The *Yersinia* high-pathogenicity island (HPI) carrying *fyuA* (encoding the pesticin receptor) and *irp* (encoding the siderophore yersiniabactin) is also present in certain non-O157 STEC lineages and was previously reported only in *stx*_2e_ carrying human isolates [19].

Domestic ruminants, especially cattle, are the major reservoirs of STEC. Other animals like sheep, goats have been confirmed as important natural reservoirs in some countries [2,20-22]. Swine also play an important role as a carrier of this pathogen. STEC strains that produce Stx2e can cause edema disease in pigs [23] and can also been isolated from human stools at low frequency. STEC carried by healthy pigs may pose a potential risk to humans [24-27]. Relatively little is known about the prevalence and characteristics of STEC in pigs in China. In this study, we isolated and characterized STEC from different pig slaughter houses and pig farms from 3 geographical regions, Beijing city, Chongqing city and Guizhou province in China.

## Results

### Prevalence of STEC in swine samples

Out of 1003 swine samples collected in this study, 25.42% (255/1003) were *stx-*positive by PCR. A total of 93 STEC isolates was obtained from 62 samples, giving a culture positive rate of 24.31% (62/255) of all *stx*-positive samples. Different *stx*-positive rates in small intestine contents (10.83%), colon contents (47.24%) and feces (19.33%) samples were observed. The colon contents samples gave the highest *stx*-positive rate (*P* < 0.05) and also the highest culture positive rate (18.09%) (*P* < 0.05) (Table 1).

**Table 1:** Prevalence of STEC in swine samples

**Sample location (city/province)**	**No. of samples**	**Type of samples (N, %)**	***stx *** **positive samples (N, %)**	**Samples with STEC isolates (N, %)**	**STEC isolates (N, %)**
Beijing	523	SC (248, 24.73)	SC (30, 8.55)	SC (3, 0.85)	SC (7, 1.99)
		CC (275, 27.42)	CC (139, 42.64)	CC (36, 11.04)	CC (57, 17.48)
Chongqing	326	F (326, 32.50)	F (63, 19.33)	F (17, 5.21)	F (23, 7.06)
Guizhou	154	SC (103, 10.27)	SC (8, 2.28)	SC (4, 1.14)	SC (4, 1.14)
		CC (51, 5.08)	CC (15, 4.60)	CC (2, 0.61)	CC (2, 0.61)
Total	1003	SC (351, 35.00)	SC (38, 10.83)	SC (7, 1.99)	SC (11, 3.13)
		CC (326, 32.50)	CC (154, 47.24)	CC (38, 11.66)	CC (59, 18.09)
		F (326, 32.50)	F (63, 19.33)	F (17, 5.21)	F (23, 7.06)

Sample codes: F, fecal samples; CC, colon contents samples; SC, small intestine contents samples. The number (N) and rate (%) are showed in the parentheses.

Only a single isolate was recovered from 44 *stx*-positive samples each. But 2 isolates per sample were recovered from 15 samples, 3 isolates per sample from 3 samples, 4 isolates per sample from 2 samples and 5 isolates per sample from 1 sample.

### Serogroups and serotypes

The 93 STEC isolates were typed into 19 serotypes, comprising 12 O serogroups and 15 H types. Forty-four isolates were O antigen untypable and 21 isolates were non motile which were designated as [H]. Nineteen serotypes were found including O2:H32/[H32], O9:H30/[H30], O20:H30/[H30], O20:H26, O76:H25, O86:H11, O87:H10, O100:H20/[H20], O114:[H30], O116:H11, O143:H38/[H38], O159:H16, O172:H30/[H30], ONT:H7, ONT:H17, ONT:H19/[H19], ONT:H21/[H21], ONT:H30/[H30], ONT:[H33].

The predominant serotypes were O20:H30/[H30], ONT:H30/[H30], O2:H32/[H32], O100:H20/[H20], O9:H30/[H30], ONT:H19/[H19], O143:H38/[H38], O172:H30/[H30] which consisted of 22 (23.66%), 22 (23.66%), 11 (11.83%), 8 (8.60%), 4 (4.30%), 4 (4.30%), 3 (3.23%) and 3 (3.23%) isolates respectively. Five serotypes (O20:H26, O86:H11, ONT:H7, ONT:H17, ONT:H21/[H21]) contained 2 isolates each and 6 serotypes (O76:H25, O87:H10, O114:[H30], O116:H11, O159:H16, ONT:[H33]) contained only 1 isolate each (Table 2).

**Table 2:** Serotypes, virulence factors and sequence types (STs) of swine STEC isolates

**ST**	**No. of isolates**	**Serotype**^**a**^	***stx***_**2e**_^**b**^	***hlyA***	***ehxA***	***astA***	***irp2***	***fyuA***	***paa***	**F18**
ST10	2	O2:H32/[H32](1CC, 1SC)	+	-	-	-	-	-	-	-
ST88	4	ONT:H19/[H19](1SC, 3CC)	+	-	-	+	+	+	-	-
ST206	3	O143:H38/[H38](3CC)	+	-	-	-	-	-	-	-
ST361	1	O20:H30 (1CC)	+	-	-	+	-	-	-	-
	1	ONT:H30 (1CC)	+	-	-	+	-	-	-	-
ST501	2	O86:H11 (2CC)	+	+	-	+	-	-	-	+
ST540	1	ONT:H30 (1SC)	+	-	-	-	-	-	-	-
	3	ONT:[H30] ( 1SC, 2CC)	+	-	-	-	-	-	-	-
	1	O114:[H30] (1CC)	+	-	-	-	-	-	-	-
ST641	1	O87:H10 (1SC)	+	+	-	-	-	-	-	+
ST694	1	ONT:[H33] (1CC)	+	-	-	+	-	-	-	-
ST710	2	O20:H26 (2 F)	+	-	-	+	-	-	-	-
	17	O20:H30/[H30](4 F, 13CC)	+	-	-	+	-	-	-	-
	1	O20:[H30] (1 F)	+	-	+	+	-	-	+	-
	3	O20:[H30](1 F, 2CC)	+	-	-	+	-	-	-	-
	3	O172:H30/[H30](3CC)	+	-	-	+	-	-	-	-
ST953	2	ONT:H17 (2CC)	+	-	-	-	-	-	+	-
ST993	10	ONT:H30 (10CC)	+	-	-	-	-	-	-	-
	2	ONT:H30 (2CC)	+	-	-	+	-	-	-	-
	3	ONT:H30/[H30](2 F, 1CC)	+	-	-	-	-	-	-	-
ST1294	1	ONT:H30 (1CC)	+	-	-	-	-	-	-	-
ST1494	2	ONT:H21/[H21](2CC)	+	-	-	+	-	-	-	-
ST2514	1	O100:H20 (1 F)	+	-	-	+	-	-	-	-
	1	O100:H20 (1SC)	+	-	-	+	-	-	+	-
	5	O100:H20/[H20](1 F,4CC)	+	-	-	-	-	-	-	-
	1	O100:[H20] (1CC)	+	-	+	-	-	-	+	-
ST3628	9	O2:H32/[H32](9 F)	+	+	-	-	-	-	-	-
ST3629	4	O9:H30/[H30](4CC)	+	-	-	+	-	-	-	-
	1	ONT:H30 (1CC)	+	-	-	+	-	-	-	-
ST3630	1	O159:H16 (1CC)	-	-	-	+	-	-	+	-
ST3633	1	O76:H25 (1 F)	+	+	-	-	-	-	-	-
ST3631	1	ONT:H7 (1SC)	+	-	-	+	-	-	+	-
ST3634	1	ONT:H7 (1SC)	+	-	-	+	-	-	-	-
ST3870	1	O116:H11(1 F)	+	+	-	+	-	-	-	+
Total	93	93	93	14	2	50	4	4	7	4

^a^The numbers and sources are showed in the parentheses. F, fecal samples; CC, colon contents samples; SC, small intestine contents samples. ONT, Not typeable with available O antisera. The H types of non-motility isolates are determined by *fliC* sequencing and indicated in the square brackets.

^b^Ninety-two STEC isolates were subtyped by primer-specific PCR except one isolate of O159:H16.

### Sorbitol fermentation and hemolysis

Out of the 93 STEC isolates, 53 (56.99%) were sorbitol-positive, covering all three types of samples and three regions. Twelve serotypes including O2:H32/[H32], O9:H30/[H30], O20:H26, O76:H25, O86:H11, O87:H10, O114:[H30], O116:H11, ONT:H17, ONT:H19/[H19], ONT:H21/[H21], ONT:[H33] were sorbitol-positive while 6 serotypes (O20:H30/[H30], O100:H20/[H20], O143:H38/[H38], O159:H16, O172:H30/[H30], ONT:H7) were sorbitol negative. All except 1 ONT:H30/[H30] isolate was sorbitol-positive.

Fourteen isolates displayed apparent β-hemolytic activity on sheep blood agar including 9 of the 11 O2:H32/[H32] isolates and 2 of the 11 O86:H11 isolates, and the single O76:H25, O87:H10 and O116:H11 isolates, the majority of which (11 isolates) were recovered from swine feces in Chongqing city. The 2 hemolytic O86:H11 isolates were isolated from colon contents in a slaughter house in Beijing city and the single O87:H10 isolate was isolated from a small intestine content in a slaughter house in Guizhou province.

### Shiga toxin genes, adhesin genes and putative virulence genes

The 93 STEC isolates were tested positive for *stx*_2_ only. All except 1 isolate was *stx*_2e_ subtype by PCR subtyping. The exception was an O159:H16 isolate which was found to carry a new variant of *stx*_2e_ by sequencing. The new variant differs from the closest *stx*_2e_ (GenBank: AM904726) by 4.51% at nucleotide level.

Three virulence-related genes (*astA*, *ehxA* and *hlyA*) and 2 markers for HPI (*irp2* and *fyuA*) were screened. 53.76% (50/93) STEC isolates carried *astA*, 15.05% (14/93) isolates contained hemolysin gene *hlyA* and only 2.15% (2/93) isolates contained enterohemolysin gene *ehxA*. All *hlyA* positive STEC isolates showed hemolytic activity on standard sheep blood agar. Hemolysis was not observed in the 2 *ehxA-*positve STEC isolates. The *irp2* and *fyuA* genes were identified in 4 STEC isolates, all of which were ONT:H19/[H19] serotypes (Table 2).

Among the 15 adherence-associated genes, 13 (*eae*, *efa1*, *iha*, *lpfA*_O113_, *lpfA*_O157/OI-154_, *lpfA*_O157/OI-141_, *toxB*, *saa*, F4, F5, F6, F17 or F41) were not detected in the 93 STEC isolates. *paa* was present in 7 STEC isolates. Two O86:H11 isolates, 1 O87:H10 isolate and 1 O116:H11 isolate carried F18. Eighty-two STEC isolates did not carry any of the adherence-associated genes tested (Table 2).

### Antibiotic resistance in the swine STEC isolates

Antimicrobial resistance was determined against 23 antibiotics. The highest prevalence was tetracycline resistance with a rate of 79.57%. Most isolates were resistant to nalidixic acid and trimethoprim-sulfamethoxazole, followed by resistance to kanamycin with a rate of 78.49%, 73.12% and 55.91% respectively. Resistance rate to streptomycin, chloramphenicol, ampicillin and piperacillin was 48.39%, 37.63%, 25.81% and 20.43%, respectively. Lower resistance was observed for cephalothin, nitrofurantoin, ciprofloxacin, ceftriaxone, aztreonam, cefotaxime, cefuroxime, gentamicin, norfloxacin, levofloxacin, ampicillin-sulbactam with a rate ranging from 2.15% to 17.20%. All isolates were susceptible to imipenem and meropenem (Additional file 1: Table S1).

**Figure 1:**
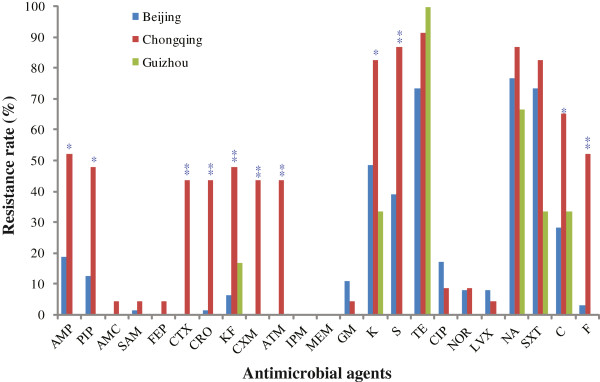
**Antimicrobial resistance profiles of the STEC isolates.** Three regions sampled are Beijing city (in blue), Chongqing city (in red), Guizhou province (in green). Statistical test was only performed between Chongqing and Beijing. A *and** were placed above the histogram for Chonqing samples if *P* < 0.05 and *P* < 0.001 respectively. Antibiotics abbreviations are: AMP, Ampicillin; PIP, Piperacillin; AMC, Amoxicillin-clavulanic acid; SAM, Ampicillin-sulbactam; FEP, Cefepime; CTX, Cefotaxime; CRO, Ceftriaxone; KF, Cephalothin; CXM, Cefuroxime; ATM, Aztreonam; IPM, Imipenem; MEM, Meropenem; GM, Gentamicin; K, Kanamycin; S, Streptomycin; TE, Tetracycline; CIP, Ciprofloxacin; NOR, Norfloxacin; LVX, Levofloxacin; NA, Nalidixic acid; SXT, Trimethoprim-sulfamethoxazole; C, Chloramphenicol; F, Nitrofurantoin.

Four isolates (4.3%) were susceptible to all 23 antimicrobial agents tested. Thirteen isolates (13.98%) were only resistant to 1 antimicrobial substance, while 76 isolates (81.72%) exhibited resistance to 2 or more antimicrobials tested. The STEC isolated from pig farms in Chongqing city showed resistance to a larger number of antimicrobial agents, and at a significantly higher rate than those isolated from slaughter houses in Beijing city (*P* < 0.05) (Figure 1 and Additional file 1: Table S1). An O116:H11 isolate exhibited multi-drug resistant phenotype against 19 of all 23 antimicrobial agents (excluding imipenem, meropenem, gentamicin and levofloxacin).

### Pulsed-field gel electrophoresis (PFGE)

**Figure 2:**
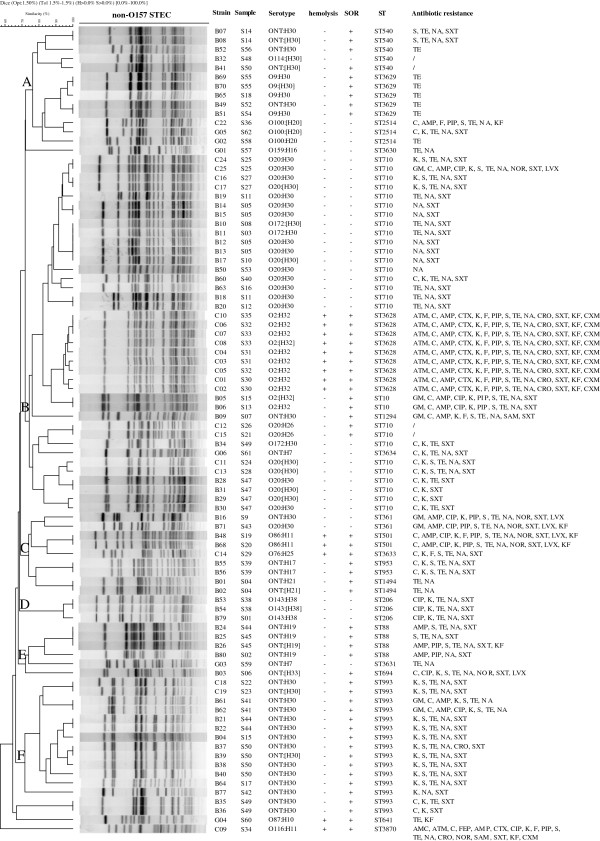
**Dendrogram of PFGE profiles of 88 STEC isolates from pigs in farms and slaughter houses.** The 6 PFGE clusters were marked on the node as **A** to **F**. Non-typeable with available O antisera was marked ONT and non-motile isolates were labeled with the H types in square brackets. Displayed on the right hand side are strain name, sample name, serotype, hemolysis, sorbitol fermentation (SOR), sequence type (ST) and antibiotic resistance. Abbreviations for antibiotics are: AMP, Ampicillin; PIP, Piperacillin; AMC, Amoxicillin-clavulanic acid; SAM, Ampicillin-sulbactam; FEP, Cefepime; CTX, Cefotaxime; CRO, Ceftriaxone; KF, Cephalothin; CXM, Cefuroxime; ATM, Aztreonam; IPM, Imipenem; MEM, Meropenem; GM, Gentamicin; K, Kanamycin; S, Streptomycin; TE, Tetracycline; CIP, Ciprofloxacin; NOR, Norfloxacin; LVX, Levofloxacin; NA, Nalidixic acid; SXT, Trimethoprim-sulfamethoxazole; C, Chloramphenicol; F, Nitrofurantoin. Place of isolates were contained in the first letter of strain names: B means Beijing city, C means Chongqing city and G means Guizhou province.

All 93 STEC isolates were analyzed by PFGE but only 88 isolates produced clear bands to give a PFGE profile which were divided into 63 PFGE patterns (EZKX01001 to EZKX01063). The most prevalent serotype O20:H30/[H30] with 22 isolates were typed into 16 PFGE patterns and the 11 O2:H32/[H32] isolates were typed into 8 PFGE patterns. An UPGMA dendrogram was constructed (Figure 2). The 88 STEC isolates could be divided into six clusters, A to F, at a similarity of 75% or greater. Cluster A contains all 4 O9:H30/[H30] and all 3 O100:H20/[H20] isolates. Cluster B contained the majority of O20:H30 isolates which were grouped into 3 subclusters. All the 11 of O2:H32/[H32] isolates also fell into cluster B as one subcluster. Cluster C was heterogenous containing 6 serotypes. Clusters D to F contained mostly one serotype: O143:H38/[H38], ONT:H19/[H19], ONT:H30/[H30] respectively. Although isolates were largely grouped together by serotypes, identical PFGE profiles were also found among isolates of different serotypes (O20:H30/[H30] and O172:H30/[H30]) which were not from the same sample but from the same sampling point.

### Multi-locus sequence typing (MLST)

The 93 STEC isolates were typed into 21 sequence types (STs) with 7 novel STs (Table 2). Four new STs (ST3628, ST3629, ST3633 and ST3634) were resulted from a novel allele in *fumC* (allele 470), *gyrB* (allele 351), *icd* (allele 396) and *recA* (allele 267) respectively. Three new STs (ST3630, ST3631 and ST3870) were due to new combinations of previously known alleles. The predominant STs were ST710 and ST993 containing 25 (26.88%) and 15 (16.13%) isolates respectively. Six STs contained 3 or more isolates with ST3628, ST2514, ST540, ST3629, ST88 and ST206 comprising 9 (9.68%), 8 (8.60%), 6 (6.45%), 5 (5.38%), 4 (4.30%) and 3 (3.23%) isolates respectively. Five STs (ST10, ST361, ST1494, ST953 and ST501) contained 2 isolates each. Eight STs (ST641, ST691, ST1294, ST3630, ST3631, ST3633, ST3634 and ST3870) had only 1 isolate each. STEC isolates from Beijing, Chongqing and Guizhou were typed into 14, 6 and 5 STs respectively. ST2514 were recovered from all 3 regions and ST710 and ST993 were recovered from 2 regions, while other STs was only found in one region.

**Figure 3:**
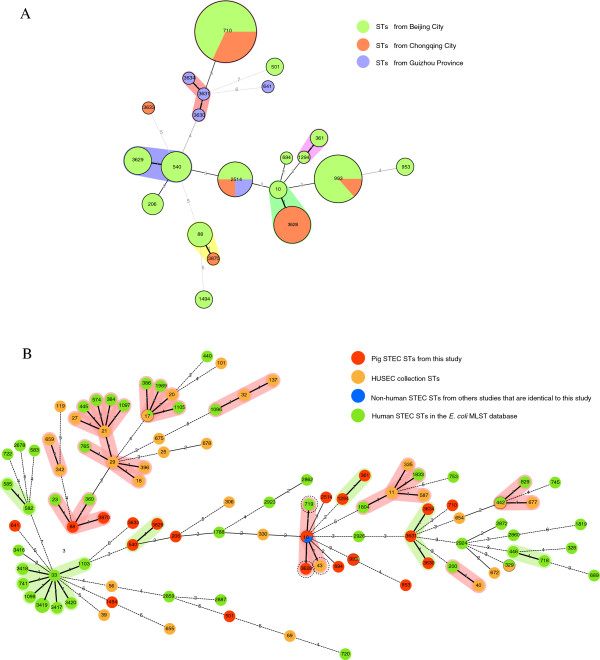
**Genetic relationships of STEC isolates based on MLST. A)** Genetic relationships of STEC sequence types (STs) from this study. Each circle represents a given ST with size proportional to the number of isolates. The colors for the slices of the pie represent places of isolates: Beijing city in green, Chongqing city in red and Guizhou province in purple. The numbers on connecting lines show the number of allelic difference between two STs. The number in a circle is the ST number. **B)** Minimal spanning tree of STs from this study, STs from the HUSEC collection and other human STEC STs. Ninety-three pig STEC isolates (in red) were compared to STs of HUSEC collection (in orange), human STEC STs (in green) and STs from other source that are identical to STs in our study (in blue) in *E. coli* MLST database. Each circle represents a given ST with the pie proportional to number of isolates in a given ST from different sources. The numbers on connecting lines show the number of allelic difference between two STs. The number in a circle is the ST number.

A minimum spanning tree was constructed (Figure 3A). Most STs differed from each other by 2 or more alleles while three pairs of STs (ST10 and ST3628, ST540 and ST3629, and ST88 and ST3870) and one set of 3 STs (ST3630, ST3631 and ST3634) differed from each other by only 1 allele. There is good concordance between STs and serotype. One ST consisted of solely or predominantly one serotype. However ST710, the most frequent ST, contained 3 serotypes, O20:H30/[H30], O172:H30/[H30] and O20:H26 with the first serotype being predominant. PFGE and MLST were also largely consistent in the clustering of the isolates (Figure 2). ST540 and ST3629 with 1 SNP difference in *icd* allele were grouped together with ST2514 in PFGE cluster A. All ST710 isolates were grouped into 2 subclusters within PFGE cluster B which were separated by ST3628, ST10 and ST1294. ST10 and ST3628 isolates were grouped together which differed by 1 SNP difference in *gyrB*. PFGE clusters D and F were inclusive of all ST206 isolates and ST993 isolates respectively. However, the 5 STs (ST361, ST501, ST953, ST1494 and ST3633) within PFGE cluster C and the 3 STs (ST88, ST3631 and ST694) within PFGE cluster E were not closely related to each other by MLST (Figure 3A). On the other hand, ST88 was not grouped together with ST3870 by PFGE, which differed by 1 SNP difference in *gyrB*. The sole ST3870 isolate C09 also differed from the 4 ST88 isolates by serotype, hemolysis and antibiotic resistance profile.

Isolates of the same STs generally showed the same or similar drug resistance patterns (Figure 2). All ST3628 isolates showed the same multi-drug resistance to 14 antibiotics. Similarly, isolates of ST206, ST953and ST1494 showed respective identical resistance profiles. All ST3629 isolates were resistant to tetracycline. However there existed variations of drug resistance within an ST. ST710 showed the most variability with resistance to 1 to 11 drugs. ST2514 which was isolated from all 3 regions also showed varied resistance profiles.

## Discussion

Different prevalence of STEC in pigs were reported previously [24,25,27-29]. Kaufmann *et al. *[24] compared the STEC shedding rate in pigs at slaughter, which varied widely and ranged from 2.1% to 70% depending on the health conditions of the pigs and the detection method used. As shown in this study the anatomic sites sampled also affected the rate of isolation and consequently affected the prevalence in the population reported. Fecal samples were commonly used [24-26]. In our study we sampled the small intestinal content, the colon content and the feces. The prevalence of STEC in the colon (47.24%) was almost 2.5 times higher than in feces (19.33%) (*P* < 0.05) and 4.4 times higher than in the small intestine (10.83%) (*P* < 0.05). STEC strains are thought to mostly colonize the colons of humans [30] and it is likely to be the same for pigs.

In this study, 93 isolates were recovered from 62 of the 255 *stx*-positive samples, giving a culture positve rate of 24.31%, this result is similar to that of Botteldoorn *et al.*[28], in which STEC isolates were obtained from 31% of the *stx* PCR-positive pig samples. Failure to isolate STEC from the *stx*-positive samples may due to the perturbation of high levels of background microflora, the loss of Stx prophages during subculture, the presence of other bacteria carrying *stx* or low levels of STEC in the samples.

In the present study, 12 serogroups and 19 serotypes were identified. The majority of these serotypes have been isolated from swine, sheep, cattle, food, and water in other countries [24,31-36]. The most prevalent serotype is O20:H30/[H30], which was also reported in cattle and sheep in different countries [31,32]. Six serotypes (O100:H20/[H20], O143:H38/[H38], O87:H10, O172:H30/[H30], O159:H16, O9:H30/[H30]) were rarely found in STEC isolates isolated from swine and other ruminants, implying that these serotypes may be restricted to the swine populations in these regions and their environments. Serotypes O86:H11, O20:NM, O100:NM, O9:NM, O172:NM and O114:NM have previously been described among STEC isolated from human patients [37-42]. Serotype O157:H7, which is common serotype causing human disease in some countries, was not detected.

A possible reason for no isolation of O157:H7 might be the method used. Isolation of O157 STEC often requires more targeted methods, such as the use of O157 immunomagnetic beads to capture the bacteria from enrichment broth and then culture on selective media [43]. We previously used immunomagnatic separation to successfully isolate O157 STEC from pigs, although that was in an outbreak setting and was in a different geographic region [44]. In this study we used CHROMagar™ ECC only and didn’t specifically target O157 STEC. CHROMagar™ ECC has been used by others for isolation of STEC from pigs [45]. However, that study did not isolate O157 STEC either. Therefore, the CHROMagar™ ECC may not be an ideal media for O157 STEC isolation.

We used sorbitol-MacConkey agar as a quick method to pick potential O157 colonies since sorbitol fermentation is a traditional feature for differentiating O157:H7 which is sorbitol-negative although there are sorbitol-positive O157 STEC [46]. In this study, a fair proportion (43%) of non-O157 STEC is actually sorbitol-negative. Therefore sorbitol fermentation is not a good indicator for O157:H7.

We analyzed multiple colonies from 21 samples to determine diversity within a sample (Figure 2). Two samples contained isolates with identical properties, suggesting they are the same strain, while the majority of the samples contain isolates belonging to the same sequence type but differing by one or more of the phenotypic or genetic properties tested, indicating that they are variants of the same clone. The most common variations are non-expression of the H antigen, variation of antibiotic resistance and/or variation in PFGE patterns. However 4 samples contained 2 different STs. Samples S15, S41, S49 and S50 all contain the prevalent ST993 and an additional ST, being ST10, ST88, ST710 and ST540 respectively, suggesting 2 different clones infecting the same pig.

Many studies have underlined the potential key role of the Stx2 subtypes in the severity of disease. Although Stx2e is not a potent subtype [47], strains harboring Stx2e have been isolated from patients with diarrhea [48]. Intimin contributes to the development of A/E lesions and is a key virulence for some STEC serotypes [49], while *ehxA* can be found in many STEC serotypes, such as O157:H7 and O26:H11 that are associated with diarrheal disease and HUS [7,50]. However, Sonntag *et al*. reported that the *stx*_2e_-positive *E. coli* isolated from healthy pigs rarely contains genes for intimin and enterohemolysin [19]. The prevalence of *ehxA* is very low in our samples at 2.15%, consistent with the findings of Sonntag *et al*. [19].

Other virulence factors may contribute to the pathogenicity of STEC. Although the role of EAST1 toxin in virulence to pigs has not been clearly determined, several studies have shown that *astA* gene is widely present among STEC isolates from both diarrheal and healthy pigs [15,24,26]. *astA* gene was also the most prevalent virulent gene (53.76%) among the 20 virulence genes tested in our study.

HPI was originally identified in *Yersinia* and now found in a range of pathogens [51], including the HUS-associated *E. coli* HUSEC041 [52] and the 2011 German HUS outbreak strain O104:H4 [53]. HPI had previously been detected in Stx2e- producing STEC strains from humans only [19]. In this study we found 4 *stx*_2e_ STEC isolates, all ONT:H19/[H19], harbored the 2 HPI genes *fyuA* and *irp* although the frequency is low at 4.3%.

Fimbrial adhesins play an important role in colonization of the pig intestine and STEC strains may express up to 5 antigenically distinct fimbrial adhesins, F4, F5, F6, F18 and F41 [18]. Different types of fimbriae can be associated with STEC diarrhea in animals of different ages [15-18]. In this study, only 4 isolates contained a fimbrial adhesin (F18). None of the other fimbrial adhesins (F4, F5, F6, F17 and F41) was detected. Of the nonfimbrial adhesin-encoding genes, *paa* was found in 7 isolates (7.5%), but *efa1*, *toxB*, *lpfA*_O157/OI-154_, *lpfA*_O157/OI-141_, *lpfA*_O113_ and *saa* were not detected in any of the 93 STEC isolates*.* Eighty-two STEC isolates did not carry any of the adherence-associated genes tested.

Coombes *et al*. [54] reported that non-LEE encoded T3SS effector (*nle*) genes of non-O157 STEC strains are correlated with outbreak and HUS potential in humans. It will be interesting to examine our STEC isolates for the presence of the *nle* genes in future studies.

Many non-O157 STEC isolated from humans and animals have shown resistance to multiple antimicrobials [26,55,56], including resistance to trimethoprim-sulfamethoxazole and β-lactams [56,57]. STEC isolates from swine feces in the United States show high resistance rates (>38%) to tetracycline, sulfamethoxazole and kanamycin but susceptible to nalidixic acid (resistance rate 0.5%) [26]. In our study, we found that only 1 of the 12 categories of antimicrobial resistance types (carbapenems) and 2 of the 23 antimicrobial agents (imipenem and meropenem) were active against all the STEC isolates. The high prevalence (>50%) of resistance to tetracycline, trimethoprim-sulfamethoxazole, nalidixic acid and kanamycin is similar to that of other studies in China [55,58]. In a study [55] of STEC from diseased pigs in Guangdong province, China, the majority of the isolates (95%) were resistant to more than 3 antimicrobials and the resistance rates to chloramphenicol (89%) and streptomycin (83%) were far higher than that of our study (37.63% and 48.39%, respectively). We also found that isolates from Chongqing showed a higher rate than those from the other 2 cities in this study. It should be noted that all samples collected from Chongqing were fecal samples while those from Beijing and Guizhou were small intestinal contents and colon contents samples, which may affect resistance profiles if different *E. coli* strains have a preference for the anatomic sites. However, it is more likely that the difference reflected the presence of resistant *E. coli* strains in different regions. Chongqing was dominated by the multidrug resistant ST3628. The differences in drug resistance rates between cities may be related to the differences in the prevalence of drug resistant STs.

Comparison with STs observed in human infections gives an indication of the potential risk for human infection of the swine STEC. We constructed an MST containing our STs, the 32 STs of the HUSEC collection and 52 human STEC STs from the *E. coli* MLST database (Figure 3B). None of the 21 STs in this study was identical to any of the 32 STs of HUSEC collection [52]. We only found one ST, ST993, which was observed in human infections. When comparison was made at clonal complex level, some of our STs fell into the same clonal complex as the human STs (Figure 3B). ST10 clonal complex contained 2 of our STs (ST10 and ST3628), 1 HUSEC ST (ST43) and 1 human STEC ST (ST719) from the MLST database. However, Hauser *et al.* found that 8 of the 35 STEC STs they isolated from foods shared the same STs with HUSEC strains and were similar in their virulence gene composition [59]. Since the STECs from foods and HUSEC collection were from the same geographical region, it is likely some of the HUSEC STECs were from local sources and not globally distributed. Our STECs from pigs may cause local human infections but there is no surveillance of human STECs in the regions where we sampled the swine STECs.

## Conclusions

In conclusion, the prevalence of STEC in healthy pigs is high (25.42%) by PCR screening although only 6.18% of the swine samples yielded an STEC isolate by microbiological culture. The vast majority of isolates belonged to a limited number of serogroups and serotypes, with O20:H30/[H30] being the predominant serotype. The majority of the STEC serotypes found in this study were also reported in other countries. All 93 STEC isolates carried the pig associated *stx*_2e_ subtype. Only a small proportion of the STEC isolates harbored *hlyA*, *ehxA* and adhersin genes. Based on comparison by serotypes and sequence types with human strains and presence of virulence genes, the STEC isolated from pigs may have a low potential to cause human disease. However, further investigations are needed to assess their public health significance in causing human disease in China.

## Methods

### Sample collection

A total of 1003 samples was collected from May 2011 to August 2012, of which 326 were fecal samples collected in pig farms in Chongqing city, 351 were small intestinal contents and 326 were colon contents collected in pig slaughter houses in Beijing city and Guizhou province. Samples were transported as soon as possible to the laboratory in the National Institute for Communicable Disease Control and Prevention, Chinese Center for Disease Control and Prevention in ice-bags cold conditions for the isolation of STEC.

### Isolation of STEC

One gram of each sample was enriched in 5 ml of modified Tryptone Soya Broth (mTSB) supplemented with novobiocin (10 mg/liter) (Oxoid, UK) and incubated at 37°C for 18 to 24 h with shaking at 200 rpm. Briefly, 150 μl of the lysis buffer (100 mM NaCl, 10 mM Tris–HCl [pH 8.3], 1 mM EDTA [pH 9.0], 1% Triton X-100) were added to the centrifuged enrichment sample, boiled for 10 min and centrifuged. The supernatant was used as template to test for the presence of *stx*_1_ and *stx*_2_ by TaqMan duplex real time PCR assay developed by Bai *et al*. [60]. One loopful of the *stx*-positive enrichment culture was directly streaked onto CHROMagar™ ECC plate (CHROMagar, Microbiology, Paris, France). After overnight incubation at 37°C, 10 blue or colorless, round moist presumptive colonies on each plate were initially picked randomly to test for the presence of *stx*_1_ and *stx*_2_ by conventional duplex PCR assay (primers listed in Table 3) and another 10 colonies were picked if the initial 10 were negative for any of the *stx* genes. The *stx*-positive colonies were plated onto Luria-Bertani (LB) plates and incubated overnight for further identification. One to 5 *stx*-positive isolates from each sample were collected for further investigation.

**Table 3:** PCR primers used for the detection of STEC virulence or adherence genes

**Targets**	**Primer**	**Oligonucleotide sequence (5′-3′)**	**Amplicon size (bp)**	**Reference**
*stx*_1_	*stx*_*1*_*-*F	AAATCGCCATTCGTTGACTACTTCT	370	[61]
	*stx*_*1*_-R	TGCCATTCTGGCAACTCGCGATGCA		
*stx*_2_	*stx*_*2*_-F	CAGTCGTCACTCACTGGTTTCATCA	283	[61]
	*stx*_*2*_-R	GGATATTCTCCCCACTCTGACACC		
*stx*_2e_	*stx*_*2e*_-F	CGGAGTATCGGGGAGAGGC	411	[62]
	*stx*_*2e*_-R	CTTCCTGACACCTTCACAGTAAAGGT		
SLT-II	GK1	ATGAAGTGTATATTATTTAAATGG	1241	[63]
	GK4	TCAGTCATTATTAAACTGCAC		
*ehxA*	*ehxA*-F	GGTGCAGCAGAAAAAGTTGTAG	1551	[64]
	*ehxA*-R	TCTCGCCTGATAGTGTTTGGTA		
*hlyA*	*hlyA*1-F	GACAAAGCACGAAAGATG	2930	[6]
	*hlyA*2-R	CAACTGCAATAAAGAAGC		
*astA*	EAST11a	CCATCAACACAGTATATCCGA	111	[65]
	EAST11b	GGTCGCGAGTGACGGCTTTGT		
*irp2*	*irp2-*F	AAGGATTCGCTGTTACCGGAC	280	[66]
	*irp2-*R	TCGTCGGGCAGCGTTTCTTCT		
*fyuA*	*fyuA-*F	TGATTAACCCCGCGACGGGAA	880	[66]
	*fyuA-*R	CGCAGTAGGCACGATGTTGTA		
*eae*	*eae-F*	ACGTTGCAGCATGGGTAACTC	815	[36]
	*eae-R*	GATCGGCAACAGTTTCACCTG		
*paa*	M155-F1	ATGAGGAAACATAATGGCAGG	350	[67]
	M155-R1	TCTGGTCAGGTCGTCAATAC		
*iha*	*iha-*F	CAGTTCAGTTTCGCATTCACC	1305	[68]
	*iha-*R	GTATGGCTCTGATGCGATG		
*saa*	*saa-*F	CGTGATGAACAGGCTATTGC	119	[14]
	*saa-*R	ATGGACATGCCTGTGGCAAC		
*toxB*	*toxB-*F	ATACCTACCTGCTCTGGATTGA	602	[69]
	*toxB-*R	TTCTTACCTGATCTGATGCAGC		
*efa1*	*efa1-*F	GAGACTGCCAGAGAAAG	479	[11]
	*efa1-*R	GGTATTGTTGCATGTTCAG		
*lpfA*_O157/OI-154_	*lpfA*_O157/OI-154_-F	GCAGGTCACCTACAGGCGGC	525	[14]
	*lpfA*_O157/OI-154_-R	CTGCGAGTCGGCGTTAGCTG		
*lpfA*_O157/OI-141_	*lpfA*_O157/OI-141_-F	CTGCGCATTGCCGTAAC	412	[70]
	*lpfA*_O157/OI-141_-R	ATTTACAGGCGAGATCGTG		
*lpfA*_O113_	*lpfA*_O113_-F	ATGAAGCGTAATATTATAG	573	[9]
	*lpfA*_O113_-R	TTATTTCTTATATTCGAC		
F4(K88)	F4-F	GCTGCATCTGCTGCATCTGGTATGG	792	[15]
	F4-R	CCACTGAGTGCTGGTAGTTACAGCC		
F5(K99)	F5-F	TGCGACTACCAATGCTTCTG	450	[15]
	F5-R	TATCCACCATTAGACGGAGC		
F6(P987)	F6-F	TCTGCTCTTAAAGCTACTGG	333	[15]
	F6-R	AACTCCACCGTTTGTATCAG		
F17	F17-F	GGGCTGACAGAGGAGGTGGGGC	411	[15]
	F17-R	CCCGGCGACAACTTCATCACCGG		
F18	F18-F	GTGAAAAGACTAGTGTTTATTTC	510	[15]
	F18-R	CTTGTAAGTAACCGCGTAAGC		
F41	F41-F	GAGGGACTTTCATCTTTTAG	431	[15]
	F41-R	AGTCCATTCCATTTATAGGC		

### Biochemical tests and serotyping of STEC isolates

All *stx*-containing isolates were confirmed to be *E. coli* by using API 20E biochemical test strips (bioMérieux, Lyon, France). Sorbitol fermentation characteristic was examined by using sorbitol-MacConkey agar (SMAC) (Oxoid, UK).

The hemolytic activity was tested by using sheep blood agar (Oxoid, UK). The presence of transparent zones around the colonies was interpreted as positive hemolytic activity [71].

The determination of O antigens was firstly carried out by testing for specific *E. coli* O groups of interest, targeting group specific genes within the O-antigen gene cluster described by DebRoy *et al.*[72]. The entire coding sequence of the *fliC* gene was amplified by PCR with the primers fliC-F (5′-ATGGCACAAGTCATTAATACCCAAC-3′) and fliC-R (5′-CTAACCCTGCAGCAGAGACA-3′) reported by Fields *et al. *[73], and then sequenced to determine the H type of each isolate. *In vitro* motility was determined by inoculation of each isolate in the center of motility agar plates (0.3% LB agar) at 37°C for up to 48 h [74]. Bacterial motility was assessed by examining the swimming ring. The O:H serotype was confirmed by the O antisera and the H antisera obtained from the Statens Serum Institut (Copenhagen, Denmark).

### *stx* subtyping

*E. coli* isolates were cultured in LB broth at 37º C for 18–24 h. DNA was extracted using Wizard Genomic DNA Purification kits (Promega, USA). The presence of Shiga toxin genes were assessed in all isolates by PCR using primers targeting the *stx*_1_ and *stx*_2_ genes (Table 3) as described by Brian *et al.*[61]. The *stx*_2_ subtypes were determined by the PCR-based subtyping method devised by Scheutz *et al*. [62]. The complete *stx*_2_ gene from a selected set of STEC isolates was amplified using primers GK1 and GK2 from Gunzer *et al. *[63] and sequenced to verify the PCR-based subtyping results. The neighbor-joining cluster analysis was employed to assign new subtypes or variants as mentioned by Scheutz *et al*. [62].

### Identification of virulence and adherence factors

All STEC isolates were tested by PCR to investigate the presence of *astA*, hemolysis related genes (*ehxA* and *hlyA*), HPI genes (*fyuA* and *irp*) and adhesion-related genes (*eae*, *paa*, *efa1*, *toxB*, *lpfA*_O157/OI-154_, *lpfA*_O157/OI-141_, *lpfA*_O113_, *saa*, F4, F5, F6, F17, F18 and F41) using the primers listed in Table 3.

### Antimicrobial susceptibility testing

Antimicrobial resistance was determined by the disc diffusion method [75]. Twelve antimicrobial groups covering 23 antimicrobial agents including penicillins (ampicillin and piperacillin), β-lactam/β-lactamase inhibitor combinations (amoxicillin-clavulanic acid and ampicillin-sulbactam), cephems (parenteral) (cephalosporins I, II, III, and IV, cefepime, cefotaxime, ceftriaxone, cephalothin and cefuroxime), monobactams (aztreonam), carbapenems (imipenem and meropenem), aminoglycosides (gentamicin, kanamycin and streptomycin), tetracyclines (tetracycline), fluoroquinolones (ciprofloxacin, norfloxacin and levofloxacin), quinolones (nalidixic acid), folate pathway inhibitors (trimethoprim-sulfamethoxazole), phenicols (chloramphenicol) and nitrofurans (nitrofurantoinz) were tested. Results were interpreted using the Clinical and Laboratory Standards Institute (CLSI, 2012) breakpoints, when available. *E. coli* ATCC^R^ 25922 was used as quality control.

### PFGE and MLST

STEC isolates were digested with *Xba*I and separated by PFGE using the non-O157 STEC PulseNet protocol (http://www.pulsenetinternational.org). Gel images were converted to Tiff files and then analyzed using BioNumerics software (Applied Maths, Sint-Martens-Latem, Belgium).

MLST was performed according to the recommendations of the *E. coli* MLST website (http://mlst.ucc.ie/mlst/dbs/Ecoli) using 7 housekeeping genes (*adk*, *fumC*, *gyrB*, *icd*, *mdh*, *purA* and *recA*). Alleles and sequence types (STs) were determined following the website instructions [76]. MLST data for the HUS-associated enterohemorrhagic *E. coli* (HUSEC) collection were obtained from http://www.ehec.org[52]. All human STEC STs from the *E. coli* MLST databases were downloaded for comparison. A minimum spanning tree based on these STs was generated with BioNumerics software.

Four novel alleles, *fumC*470, *gyrB*351, *icd*396 and *recA*267 were submitted to *E. coli* MLST website. The sequences obtained in this study have been deposited in GenBank: KC924398 (*icd*396), KC924399 (*gyrB*351), KC924400 (*fumC*470), KC924401 (*recA*267) and KC339670 (a new variant of *stx*_2e_).

### Statistical analysis

Statistical tests were performed using SAS, Version 9.1 (SAS Institute Inc., Cary, NC., USA). Statistically significant differences were calculated using a χ2 test where appropriate. *P* values of <0.05 were considered statistically significant.

### Ethics statement

Samples of pig feces, small intestinal contents and colon contents of finished pig were acquired with the oral consent from the pig owners. This study was reviewed and approved by the ethics committee of the National Institute for Communicable Disease Control and Prevention, China CDC, according to the medical research regulations of the National Health and Family Planning Commission of People’s Republic of China (permit number 2011-10-4).

## Competing interests

The authors declare that they have no competing interests.

## Authors’ contributions

QM carried out the sample collection, isolation of STEC, biochemical tests and serotyping of STEC isolates, identification of virulence and adherence factors, antimicrobial susceptibility testing, MLST, *stx* subtyping, data analysis and drafting of the manuscript. YX and RL carried out study design, overseeing the study, and editing of the manuscript. The rest of the authors contributed sample collection, strains isolation, biochemical tests and serotyping of STEC isolates, MLST, or PFGE. All authors read and approved the final manuscript.

## Supplementary Material

Additional file 1: Table S1 Antibiotic resistances of swine STEC isolates. Click here for file

## References

[B1] Nataro JP Kaper JB Diarrheagenic *Escherichia coli* Clin Microbiol Rev 1998 11 1 142 201 9457432 10.1128/cmr.11.1.142 PMC121379

[B2] Griffin PM Tauxe RV The epidemiology of infections caused by *Escherichia coli* O157:H7, other enterohemorrhagic E. coli, and the associated hemolytic uremic syndrome Epidemiol Rev 1991 13 60 98 1765120 10.1093/oxfordjournals.epirev.a036079

[B3] Bettelheim KA The non-O157 shiga-toxigenic (verocytotoxigenic) *Escherichia coli*; under-rated pathogens Crit Rev Microbiol 2007 33 1 67 87 10.1080/10408410601172172 17453930

[B4] Paton JC Paton AW Pathogenesis and diagnosis of Shiga toxin-producing *Escherichia coli* infections Clin Microbiol Rev 1998 11 3 450 479 9665978 10.1128/cmr.11.3.450 PMC88891

[B5] Savarino SJ Fasano A Watson J Martin BM Levine MM Guandalini S Guerry P Enteroaggregative *Escherichia coli* heat-stable enterotoxin 1 represents another subfamily of E. coli heat-stable toxin Proc Natl Acad Sci USA 1993 90 7 3093 3097 10.1073/pnas.90.7.3093 8385356 PMC46243

[B6] Boyd EF Hartl DL Chromosomal regions specific to pathogenic isolates of *Escherichia coli* have a phylogenetically clustered distribution J Bacteriol 1998 180 5 1159 1165 9495754 10.1128/jb.180.5.1159-1165.1998 PMC107003

[B7] Cookson AL Bennett J Thomson-Carter F Attwood GT Molecular subtyping and genetic analysis of the enterohemolysin gene (ehxA) from Shiga toxin-producing *Escherichia coli* and atypical enteropathogenic E. coli Appl Environ Microbiol 2007 73 20 6360 6369 10.1128/AEM.00316-07 17720842 PMC2075064

[B8] Batisson I Guimond MP Girard F An H Zhu C Oswald E Fairbrother JM Jacques M Harel J Characterization of the novel factor paa involved in the early steps of the adhesion mechanism of attaching and effacing *Escherichia coli* Infect Immun 2003 71 8 4516 4525 10.1128/IAI.71.8.4516-4525.2003 12874331 PMC166039

[B9] Doughty S Sloan J Bennett-Wood V Robertson M Robins-Browne RM Hartland EL Identification of a novel fimbrial gene cluster related to long polar fimbriae in locus of enterocyte effacement-negative strains of enterohemorrhagic *Escherichia coli* Infect Immun 2002 70 12 6761 6769 10.1128/IAI.70.12.6761-6769.2002 12438351 PMC133005

[B10] Johnson TJ Nolan LK Pathogenomics of the virulence plasmids of *Escherichia coli* Microbiol Mol Biol Rev 2009 73 4 750 774 10.1128/MMBR.00015-09 19946140 PMC2786578

[B11] Nicholls L Grant TH Robins-Browne RM Identification of a novel genetic locus that is required for in vitro adhesion of a clinical isolate of enterohaemorrhagic *Escherichia coli* to epithelial cells Mol Microbiol 2000 35 2 275 288 10.1046/j.1365-2958.2000.01690.x 10652089

[B12] Paton AW Srimanote P Woodrow MC Paton JC Characterization of Saa, a novel autoagglutinating adhesin produced by locus of enterocyte effacement-negative Shiga-toxigenic *Escherichia coli* strains that are virulent for humans Infect Immun 2001 69 11 6999 7009 10.1128/IAI.69.11.6999-7009.2001 11598075 PMC100080

[B13] Tarr PI Bilge SS Vary JC Jr Jelacic S Habeeb RL Ward TR Baylor MR Besser TE Iha: a novel *Escherichia coli* O157:H7 adherence-conferring molecule encoded on a recently acquired chromosomal island of conserved structure Infect Immun 2000 68 3 1400 1407 10.1128/IAI.68.3.1400-1407.2000 10678953 PMC97294

[B14] Toma C Martinez Espinosa E Song T Miliwebsky E Chinen I Iyoda S Iwanaga M Rivas M Distribution of putative adhesins in different seropathotypes of Shiga toxin-producing *Escherichia coli* J Clin Microbiol 2004 42 11 4937 4946 10.1128/JCM.42.11.4937-4946.2004 15528677 PMC525252

[B15] Vu-Khac H Holoda E Pilipcinec E Blanco M Blanco JE Dahbi G Mora A Lopez C Gonzalez EA Blanco J Serotypes, virulence genes, intimin types and PFGE profiles of *Escherichia coli* isolated from piglets with diarrhoea in Slovakia Vet J 2007 174 1 176 187 10.1016/j.tvjl.2006.05.019 16956777

[B16] Toledo A Gomez D Cruz C Carreon R Lopez J Giono S Castro AM Prevalence of virulence genes in *Escherichia coli* strains isolated from piglets in the suckling and weaning period in Mexico J Med Microbiol 2012 61 Pt 1 148 156 21852524 10.1099/jmm.0.031302-0

[B17] Smeds A Pertovaara M Timonen T Pohjanvirta T Pelkonen S Palva A Mapping the binding domain of the F18 fimbrial adhesin Infect Immun 2003 71 4 2163 2172 10.1128/IAI.71.4.2163-2182.2003 12654838 PMC152074

[B18] Nagy B Fekete PZ Enterotoxigenic *Escherichia coli* (ETEC) in farm animals Vet Res 1999 30 2–3 259 284 10367358

[B19] Sonntag AK Bielaszewska M Mellmann A Dierksen N Schierack P Wieler LH Schmidt MA Karch H Shiga toxin 2e-producing *Escherichia coli* isolates from humans and pigs differ in their virulence profiles and interactions with intestinal epithelial cells Appl Environ Microbiol 2005 71 12 8855 8863 10.1128/AEM.71.12.8855-8863.2005 16332882 PMC1317431

[B20] Prendergast DM Lendrum L Pearce R Ball C McLernon J O’Grady D Scott L Fanning S Egan J Gutierrez M Verocytotoxigenic *Escherichia coli* O157 in beef and sheep abattoirs in Ireland and characterisation of isolates by Pulsed-Field Gel Electrophoresis and Multi-Locus Variable Number of Tandem Repeat Analysis Int J Food Microbiol 2011 144 3 519 527 10.1016/j.ijfoodmicro.2010.11.012 21115208

[B21] Karmali MA Gannon V Sargeant JM Verocytotoxin-producing *Escherichia coli* (VTEC) Vet Microbiol 2010 140 3–4 360 370 19410388 10.1016/j.vetmic.2009.04.011

[B22] Meng Q Xiong Y Lan R Ye C Wang T Qi T Wang Y Wang H Bai X Bai X SNP genotyping of enterohemorrhagic *Escherichia coli* O157:H7 isolates from China and genomic identity of the 1999 Xuzhou outbreak Infect Genet Evol 2013 16C 275 281 23499772 10.1016/j.meegid.2013.02.018

[B23] Weinstein DL Jackson MP Samuel JE Holmes RK O’Brien AD Cloning and sequencing of a Shiga-like toxin type II variant from *Escherichia coli* strain responsible for edema disease of swine J Bacteriol 1988 170 9 4223 4230 3045088 10.1128/jb.170.9.4223-4230.1988 PMC211431

[B24] Kaufmann M Zweifel C Blanco M Blanco JE Blanco J Beutin L Stephan R *Escherichia coli* O157 and non-O157 Shiga toxin-producing *Escherichia coli* in fecal samples of finished pigs at slaughter in Switzerland J Food Prot 2006 69 2 260 266 16496563 10.4315/0362-028x-69.2.260

[B25] Fratamico PM Bagi LK Bush EJ Solow BT Prevalence and characterization of shiga toxin-producing *Escherichia coli* in swine feces recovered in the national animal health monitoring system’s swine 2000 study Appl Environ Microbiol 2004 70 12 7173 7178 10.1128/AEM.70.12.7173-7178.2004 15574914 PMC535163

[B26] Fratamico PM Bhagwat AA Injaian L Fedorka-Cray PJ Characterization of Shiga toxin-producing *Escherichia coli* strains isolated from swine feces Foodborne Pathog Dis 2008 5 6 827 838 10.1089/fpd.2008.0147 18991545

[B27] Rios M Prado V Trucksis M Arellano C Borie C Alexandre M Fica A Levine MM Clonal diversity of Chilean isolates of enterohemorrhagic *Escherichia coli* from patients with hemolytic-uremic syndrome, asymptomatic subjects, animal reservoirs, and food products J Clin Microbiol 1999 37 3 778 781 9986852 10.1128/jcm.37.3.778-781.1999 PMC84553

[B28] Botteldoorn N Heyndrickx M Rijpens N Herman L Detection and characterization of verotoxigenic *Escherichia coli* by a VTEC/EHEC multiplex PCR in porcine faeces and pig carcass swabs Res Microbiol 2003 154 2 97 104 10.1016/S0923-2508(03)00028-7 12648724

[B29] Cardeti GF Tagliabue S Losio N Caprioli A Pacciarini ML Detection and characterization of Shiga toxin-producing E. coli (STEC) in different samples from various animal species: One year of experience 1999 University of Liège, Belgium: Proceedings of the Conference of Pathogenicity and Virulence of VTEC: 8–10 November 1999

[B30] Valdivieso-Garcia A MacLeod DL Clarke RC Gyles CL Lingwood C Boyd B Durette A Comparative cytotoxicity of purified Shiga-like toxin-IIe on porcine and bovine aortic endothelial and human colonic adenocarcinoma cells J Med Microbiol 1996 45 5 331 337 10.1099/00222615-45-5-331 8918947

[B31] Houser BA Donaldson SC Padte R Sawant AA DebRoy C Jayarao BM Assessment of phenotypic and genotypic diversity of *Escherichia coli* shed by healthy lactating dairy cattle Foodborne Pathog Dis 2008 5 1 41 51 10.1089/fpd.2007.0036 18260814

[B32] Grant MA Mogler MA Harris DL Comparison of enrichment procedures for shiga toxin-producing *Escherichia coli* in wastes from commercial swine farms J Food Prot 2009 72 9 1982 1986 19777903 10.4315/0362-028x-72.9.1982

[B33] Sanchez S Garcia-Sanchez A Martinez R Blanco J Blanco JE Blanco M Dahbi G Mora A Hermoso de Mendoza J Alonso JM Detection and characterisation of Shiga toxin-producing *Escherichia coli* other than *Escherichia coli* O157:H7 in wild ruminants Vet J 2009 180 3 384 388 10.1016/j.tvjl.2008.01.011 18337133

[B34] Beutin L Miko A Krause G Pries K Haby S Steege K Albrecht N Identification of human-pathogenic strains of Shiga toxin-producing *Escherichia coli* from food by a combination of serotyping and molecular typing of Shiga toxin genes Appl Environ Microbiol 2007 73 15 4769 4775 10.1128/AEM.00873-07 17557838 PMC1951031

[B35] Lienemann T Pitkanen T Antikainen J Molsa E Miettinen I Haukka K Vaara M Siitonen A Shiga toxin-producing *Escherichia coli* O100:H(−): *stx2e* in drinking water contaminated by waste water in Finland Curr Microbiol 2011 62 4 1239 1244 10.1007/s00284-010-9832-x 21188590

[B36] Kobayashi H Shimada J Nakazawa M Morozumi T Pohjanvirta T Pelkonen S Yamamoto K Prevalence and characteristics of shiga toxin-producing *Escherichia coli* from healthy cattle in Japan Appl Environ Microbiol 2001 67 1 484 489 10.1128/AEM.67.1.484-489.2001 11133487 PMC92607

[B37] Bower JR Congeni BL Cleary TG Stone RT Wanger A Murray BE Mathewson JJ Pickering LK *Escherichia coli* O114:nonmotile as a pathogen in an outbreak of severe diarrhea associated with a day care center J Infect Dis 1989 160 2 243 247 10.1093/infdis/160.2.243 2668422

[B38] Blanco JE Blanco M Alonso MP Mora A Dahbi G Coira MA Blanco J Serotypes, virulence genes, and intimin types of Shiga toxin (verotoxin)-producing *Escherichia coli* isolates from human patients: prevalence in Lugo, Spain, from 1992 through 1999 J Clin Microbiol 2004 42 1 311 319 10.1128/JCM.42.1.311-319.2004 14715771 PMC321739

[B39] Orth D Grif K Fisher I Fruth A Tschape H Scheutz F Dierich MP Wurzner R Emerging Shiga toxin-producing *Escherichia coli* serotypes in Europe: O100:H– and O127:H40 Curr Microbiol 2006 53 5 428 429 10.1007/s00284-006-0209-0 17066335

[B40] Kappeli U Hachler H Giezendanner N Beutin L Stephan R Human infections with non-O157 Shiga toxin-producing *Escherichia coli*, Switzerland, 2000–2009 Emerg Infect Dis 2011 17 2 180 185 10.3201/eid1702.100909 21291586 PMC3204765

[B41] Cornu G Proesmans W Dediste A Jacobs F Van De Walle J Mertens A Ramet J Lauwers S Hemolytic uremic syndrome in Belgium: incidence and association with verocytotoxin-producing *Escherichia coli* infection Clin Microbiol Infect 1999 5 1 16 22 10.1111/j.1469-0691.1999.tb00093.x 11856208

[B42] Gonzalez R Diaz C Marino M Cloralt R Pequeneze M Perez-Schael I Age-specific prevalence of *Escherichia coli* with localized and aggregative adherence in Venezuelan infants with acute diarrhea J Clin Microbiol 1997 35 5 1103 1107 9114389 10.1128/jcm.35.5.1103-1107.1997 PMC232711

[B43] Chapman PA Wright DJ Siddons CA A comparison of immunomagnetic separation and direct culture for the isolation of verocytotoxin-producing *Escherichia coli* O157 from bovine faeces J Med Microbiol 1994 40 6 424 427 10.1099/00222615-40-6-424 8006935

[B44] Xiong Y Wang P Lan R Ye C Wang H Ren J Jing H Wang Y Zhou Z Bai X A novel *Escherichia coli* O157:H7 clone causing a major hemolytic uremic syndrome outbreak in China PLoS One 2012 7 4 e36144 10.1371/journal.pone.0036144 22558360 PMC3338595

[B45] Ho WS Tan LK Ooi PT Yeo CC Thong KL Prevalence and characterization of verotoxigenic-*Escherichia coli* isolates from pigs in Malaysia BMC Vet Res 2013 9 109 10.1186/1746-6148-9-109 23731465 PMC3681573

[B46] Karch H Bielaszewska M Sorbitol-fermenting Shiga toxin-producing *Escherichia coli* O157:H(−) strains: epidemiology, phenotypic and molecular characteristics, and microbiological diagnosis J Clin Microbiol 2001 39 6 2043 2049 10.1128/JCM.39.6.2043-2049.2001 11376032 PMC88086

[B47] Fuller CA Pellino CA Flagler MJ Strasser JE Weiss AA Shiga toxin subtypes display dramatic differences in potency Infect Immun 2011 79 3 1329 1337 10.1128/IAI.01182-10 21199911 PMC3067513

[B48] Friedrich AW Bielaszewska M Zhang WL Pulz M Kuczius T Ammon A Karch H *Escherichia coli* harboring Shiga toxin 2 gene variants: frequency and association with clinical symptoms J Infect Dis 2002 185 1 74 84 10.1086/338115 11756984

[B49] Jerse AE Kaper JB The *eae* gene of enteropathogenic *Escherichia coli* encodes a 94-kilodalton membrane protein, the expression of which is influenced by the EAF plasmid Infect Immun 1991 59 12 4302 4309 1682258 10.1128/iai.59.12.4302-4309.1991 PMC259041

[B50] Zhang WL Bielaszewska M Liesegang A Tschape H Schmidt H Bitzan M Karch H Molecular characteristics and epidemiological significance of Shiga toxin-producing *Escherichia coli* O26 strains J Clin Microbiol 2000 38 6 2134 2140 10834966 10.1128/jcm.38.6.2134-2140.2000 PMC86746

[B51] Schubert S Rakin A Heesemann J The *Yersinia* high-pathogenicity island (HPI): evolutionary and functional aspects Int J Med Microbiol 2004 294 2–3 83 94 15493818 10.1016/j.ijmm.2004.06.026

[B52] Mellmann A Bielaszewska M Kock R Friedrich AW Fruth A Middendorf B Harmsen D Schmidt MA Karch H Analysis of collection of hemolytic uremic syndrome-associated enterohemorrhagic *Escherichia coli* Emerg Infect Dis 2008 14 8 1287 1290 10.3201/eid1408.071082 18680658 PMC2600372

[B53] Bielaszewska M Mellmann A Zhang W Kock R Fruth A Bauwens A Peters G Karch H Characterisation of the *Escherichia coli* strain associated with an outbreak of haemolytic uraemic syndrome in Germany, 2011: a microbiological study Lancet Infect Dis 2011 11 9 671 676 21703928 10.1016/S1473-3099(11)70165-7

[B54] Coombes BK Wickham ME Mascarenhas M Gruenheid S Finlay BB Karmali MA Molecular analysis as an aid to assess the public health risk of non-O157 Shiga toxin-producing *Escherichia coli* strains Appl Environ Microbiol 2008 74 7 2153 2160 10.1128/AEM.02566-07 18245257 PMC2292595

[B55] Wang XM Liao XP Liu SG Zhang WJ Jiang HX Zhang MJ Zhu HQ Sun Y Sun J Li AX Serotypes, virulence genes, and antimicrobial susceptibility of *Escherichia coli* isolates from pigs Foodborne Pathog Dis 2011 8 6 687 692 10.1089/fpd.2010.0739 21457048

[B56] Stephan R Schumacher S Resistance patterns of non-O157 Shiga toxin-producing *Escherichia coli* (STEC) strains isolated from animals, food and asymptomatic human carriers in Switzerland Lett Appl Microbiol 2001 32 2 114 117 10.1046/j.1472-765x.2001.00867.x 11169054

[B57] Uemura R Sueyoshi M Nagayoshi M Nagatomo H Antimicrobial susceptibilities of Shiga toxin-producing *Escherichia coli* isolates from pigs with edema disease in Japan Microbiol Immunol 2003 47 1 57 61 10.1111/j.1348-0421.2003.tb02786.x 12636254

[B58] Zhao S White DG Ge B Ayers S Friedman S English L Wagner D Gaines S Meng J Identification and characterization of integron-mediated antibiotic resistance among Shiga toxin-producing *Escherichia coli* isolates Appl Environ Microbiol 2001 67 4 1558 1564 10.1128/AEM.67.4.1558-1564.2001 11282605 PMC92769

[B59] Hauser E Mellmann A Semmler T Stoeber H Wieler LH Karch H Kuebler N Fruth A Harmsen D Weniger T Phylogenetic and molecular analysis of food-borne shiga toxin-producing *Escherichia coli* Appl Environ Microbiol 2013 79 8 2731 2740 10.1128/AEM.03552-12 23417002 PMC3623172

[B60] Bai X Zhao A Lan R Xin Y Xie H Meng Q Jin D Yu B Sun H Lu S Shiga toxin-producing *Escherichia coli* in yaks (*Bos grunniens*) from the Qinghai-Tibetan plateau, China PLoS One 2013 8 5 e65537 23776496 10.1371/journal.pone.0065537 PMC3679134

[B61] Brian MJ Frosolono M Murray BE Miranda A Lopez EL Gomez HF Cleary TG Polymerase chain reaction for diagnosis of enterohemorrhagic *Escherichia coli* infection and hemolytic-uremic syndrome J Clin Microbiol 1992 30 7 1801 1806 1629337 10.1128/jcm.30.7.1801-1806.1992 PMC265384

[B62] Scheutz F Teel LD Beutin L Pierard D Buvens G Karch H Mellmann A Caprioli A Tozzoli R Morabito S Multicenter evaluation of a sequence-based protocol for subtyping Shiga toxins and standardizing Stx nomenclature J Clin Microbiol 2012 50 9 2951 2963 10.1128/JCM.00860-12 22760050 PMC3421821

[B63] Gunzer F Bohm H Russmann H Bitzan M Aleksic S Karch H Molecular detection of sorbitol-fermenting *Escherichia coli* O157 in patients with hemolytic-uremic syndrome J Clin Microbiol 1992 30 7 1807 1810 1629338 10.1128/jcm.30.7.1807-1810.1992 PMC265385

[B64] Rey J Blanco JE Blanco M Mora A Dahbi G Alonso JM Hermoso M Hermoso J Alonso MP Usera MA Serotypes, phage types and virulence genes of shiga-producing *Escherichia coli* isolated from sheep in Spain Vet Microbiol 2003 94 1 47 56 10.1016/S0378-1135(03)00064-6 12742715

[B65] Yamamoto T Echeverria P Detection of the enteroaggregative *Escherichia coli* heat-stable enterotoxin 1 gene sequences in enterotoxigenic *E. coli* strains pathogenic for humans Infect Immun 1996 64 4 1441 1445 8606115 10.1128/iai.64.4.1441-1445.1996 PMC173940

[B66] Karch H Schubert S Zhang D Zhang W Schmidt H Olschlager T Hacker J A genomic island, termed high-pathogenicity island, is present in certain non-O157 Shiga toxin-producing *Escherichia coli* clonal lineages Infect Immun 1999 67 11 5994 6001 10531259 10.1128/iai.67.11.5994-6001.1999 PMC96985

[B67] Zweifel C Schumacher S Beutin L Blanco J Stephan R Virulence profiles of Shiga toxin 2e-producing *Escherichia coli* isolated from healthy pig at slaughter Vet Microbiol 2006 117 2–4 328 332 16872761 10.1016/j.vetmic.2006.06.017

[B68] Schmidt H Zhang WL Hemmrich U Jelacic S Brunder W Tarr PI Dobrindt U Hacker J Karch H Identification and characterization of a novel genomic island integrated at *selC* in locus of enterocyte effacement-negative, Shiga toxin-producing *Escherichia coli* Infect Immun 2001 69 11 6863 6873 10.1128/IAI.69.11.6863-6873.2001 11598060 PMC100065

[B69] Tarr CL Large TM Moeller CL Lacher DW Tarr PI Acheson DW Whittam TS Molecular characterization of a serotype O121:H19 clone, a distinct Shiga toxin-producing clone of pathogenic *Escherichia coli* Infect Immun 2002 70 12 6853 6859 10.1128/IAI.70.12.6853-6859.2002 12438362 PMC133070

[B70] Szalo IM Goffaux F Pirson V Pierard D Ball H Mainil J Presence in bovine enteropathogenic (EPEC) and enterohaemorrhagic (EHEC) *Escherichia coli* of genes encoding for putative adhesins of human EHEC strains Res Microbiol 2002 153 10 653 658 10.1016/S0923-2508(02)01379-7 12558184

[B71] Frydendahl K Prevalence of serogroups and virulence genes in *Escherichia coli* associated with postweaning diarrhoea and edema disease in pigs and a comparison of diagnostic approaches Vet Microbiol 2002 85 2 169 182 10.1016/S0378-1135(01)00504-1 11844623

[B72] DebRoy C Roberts E Fratamico PM Detection of O antigens in *Escherichia coli* Anim Health Res Rev 2011 12 2 169 185 10.1017/S1466252311000193 22152292

[B73] Fields PI Blom K Hughes HJ Helsel LO Feng P Swaminathan B Molecular characterization of the gene encoding H antigen in *Escherichia coli* and development of a PCR-restriction fragment length polymorphism test for identification of *E. coli* O157:H7 and O157:NM J Clin Microbiol 1997 35 5 1066 1070 9114382 10.1128/jcm.35.5.1066-1070.1997 PMC232704

[B74] Fontaine F Stewart EJ Lindner AB Taddei F Mutations in two global regulators lower individual mortality in *Escherichia coli* Mol Microbiol 2008 67 1 2 14 18036141 10.1111/j.1365-2958.2007.05988.x PMC2229837

[B75] CLSI Performance Standards for Antimicrobial Susceptibility Testing; Twenty-Second Informational ament 2012 Wayne, Pennsylvania: Clinical and Laboratory Standards Institute

[B76] Wirth T Falush D Lan R Colles F Mensa P Wieler LH Karch H Reeves PR Maiden MC Ochman H Sex and virulence in *Escherichia coli*: an evolutionary perspective Mol Microbiol 2006 60 5 1136 1151 10.1111/j.1365-2958.2006.05172.x 16689791 PMC1557465

