# Supplementary material for: Characterization of Shiga toxin-producing Escherichia coli isolated from healthy pigs in China
Source: BMC Microbiol. 2014 Jan 6;14:5. doi: 10.1186/1471-2180-14-5 (PMC3893481; doi:10.1186/1471-2180-14-5)
Supplement: Additional file 1: Table S1 — Antibiotic resistances of swine STEC isolates. [file 1471-2180-14-5-S1.docx]

**Additional file:**

Supplementary Table 1 -**Antibiotic resistances of swine STEC isolates**

| **Class/Antimicrobial** | **No. and percentage of Resistant isolates**^$^ | | | |
| --- | --- | --- | --- | --- |
|  | Beijing (n=64) | Chongqing (n=23) | Guizhou (n=6) | **Total (n=93)** |
| **Penicillins** | | | | |
| Ampicillin | **12(18.75%)** | **12(52.17%)** | 0(0%) | 24(25.81%) |
| Piperacillin | **8(12.5%)** | **11(47.83%)** | 0(0%) | 19(20.43%) |
| **β-lactam/ β-lactamase inhibitor combinations** | | | | |
| Amoxicillin-clavulanic acid | 0(0%) | 1(4.35%) | 0(0%) | 1(1.08%) |
| Ampicillin-sulbactam | 1(1.56%) | 1(4.35%) | 0(0%) | 2(2.15%) |
| **Cephems (parenteral) (including cephalosporins I, II, III, and IV)** | | | | |
| Cefepime | 0(0%) | 1(4.35%) | 0(0%) | 1(1.08%) |
| Cefotaxime | **0(0%)** | **10(43.48%)** | 0(0%) | 10(10.75%) |
| Ceftriaxone | **1(1.56%)** | **10(43.48%)** | 0(0%) | 11(11.83%) |
| Cephalothin | **4(6.25%)** | **11(47.83%)** | 1(16.67%) | 16(17.2%) |
| Cefuroxime | **0(0%)** | **10(43.48%)** | 0(0%) | 10(10.75%) |
| **Monobactams** | | | | |
| Aztreonam | **0(0%)** | **10(43.48%)** | 0(0%) | 10(10.75%) |
| **Carbapenems** | | | | |
| Imipenem | 0(0%) | 0(0%) | 0(0%) | 0(0%) |
| Meropenem | 0(0%) | 0(0%) | 0(0%) | 0(0%) |
| **Aminoglycosides** | | | | |
| Gentamicin | 7(10.94%) | 1(4.35%) | 0(0%) | 8(8.6%) |
| Kanamycin | **31(48.44%)** | **19(82.61%)** | 2(33.33%) | 52(55.91%) |
| Streptomycin | **25(39.06%)** | **20(86.96%)** | 0(0%) | 45(48.39%) |
| **Tetracyclines** | | | | |
| Tetracycline | 47(73.44%) | 21(91.3%) | 6(100%) | 74(79.57%) |
| **Fluoroquinolones** | | | | |
| Ciprofloxacin | 11(17.19%) | 2(8.7%) | 0(0%) | 13(13.98%) |
| Norfloxacin | 5(7.81%) | 2(8.7%) | 0(0%) | 7(7.53%) |
| Levofloxacin | 5(7.81%) | 1(4.35%) | 0(0%) | 6(6.45%) |
| **Quinolones** | | | | |
| Nalidixic acid | 49(76.56%) | 20(86.96%) | 4(66.67%) | 73(78.49%) |
| **Folate pathway inhibitors** | | | | |
| Trimethoprim-sulfamethoxazole | 47(73.44%) | 19(82.61%) | 2(33.33%) | 68(73.12%) |
| **Phenicols** | | | | |
| Chloramphenicol | **18(28.13%)** | **15(65.22%)** | 2(33.33%) | 35(37.63%) |
| **Nitrofurans** | | | | |
| Nitrofurantoin | **2(3.13%)** | **12(52.17%)** | 0(0%) | 14(15.05%) |

^$^ Statistically significant difference (*P*<0.05) between Chongqing and Beijing samples is highlighted in bold. Note that there were too few samples from Guizhou to be included for statistical test.
